# Hospital Preparations for Viral Hemorrhagic Fever Patients and Experience Gained from Admission of an Ebola Patient

**DOI:** 10.3201/eid2202.151393

**Published:** 2016-02

**Authors:** J.J. Mark Haverkort, A.L.C. (Ben) Minderhoud, Jelte D.D. Wind, Luke P.H. Leenen, Andy I.M. Hoepelman, Pauline M. Ellerbroek

**Affiliations:** University Medical Centre of Utrecht, Utrecht, the Netherlands (J.J.M. Haverkort, A.L.C. Minderhoud, L.P.H. Leenen, A.I.M. Hoepelman, P.M. Ellerbroek);; University Medical Centre of Utrecht Major Incident Hospital, Utrecht (J.J.M. Haverkort, J.D.D. Wind, L.P.H. Leenen, P.M. Ellerbroek)

**Keywords:** viral hemorrhagic fever, biosafety level, preparedness, personal protective equipment, major incident hospital, disease outbreak, Ebola virus disease, viruses, Ebola virus, the Netherlands, Ebola

## Abstract

The Major Incident Hospital of the University Medical Centre of Utrecht has a longstanding history of preparing for the management of highly pathogenic and infectious organisms. An assessment of the hospital’s preparations for an outbreak of viral hemorrhagic fever and its experience during admission of a patient with Ebola virus disease showed that the use of the buddy system, frequent training, and information sessions for staff and their relatives greatly increased the sense of safety and motivation among staff. Differing procedures among ambulance services limited the number of services used for transporting patients. Waste management was the greatest concern, and destruction of waste had to be outsourced. The admission of an Ebola patient proceeded without incident but led to considerable demands on staff. The maximum time allowed for wearing personal protective equipment was 45 minutes to ensure safety, and an additional 20 minutes was needed for recovery.

## Abstract 2

Clear protocols, a buddy system, and intensive staff training increased the sense of safety and motivation among staff.

The Ebola virus disease (EVD) epidemic during 2014–2015 led hospitals worldwide to prepare for the triage and admission of Ebola virus (EBOV)–infected patients (*1*). During the fall of 2014, the Ministry of Health, Welfare, and Sport of the Netherlands requested that the Major Incident Hospital (MIH) provide 4 beds for the admission of EBOV-infected international healthcare workers and military personnel. The MIH is a government-funded, standby facility in the basement of the University Medical Centre of Utrecht (UMC Utrecht) that provides 200 beds to ensure capacity and optimal infrastructure for the triage and care of victims of large-scale trauma, nuclear, chemical, or biological incidents (*2*). The MIH benefits from a substantial amount of resources (e.g., materials and personnel) shared with UMC Utrecht and the adjoining Central Military Hospital.

The MIH contains an isolation facility separate from other hospital infrastructure and air systems for the care of patients infected with highly pathogenic and infectious organisms (those designated as Biosafety Levels 3 and 4). This facility contains 4 isolation rooms equipped with a negative air pressure system and double air filtering. For the past 14 years, the MIH has been training staff to care for patients with viruses with aerosol transmission, and the MIH is the only center in the Netherlands designated to treat smallpox. Following the request of the Ministry of Health, Welfare, and Sport to prepare for the admission of EBOV-infected patients, all previously developed procedures were revised for the treatment of patients with viral hemorrhagic fever (VHF). We present an overview of the preparations made at the MIH in the fall of 2014, pending a possible VHF outbreak, and the experience gained from the admission of an EBOV-infected patient.

## Preparation Phase

A task group, which consisted of infection prevention experts and specialists in infectious diseases, virology, acute medicine, intensive care, pediatrics, and occupational medicine, prepared for all procedures involved in handling VHF. Other members included team leader nurses; officers for communication, security, waste management, and support services; and management representatives.

### Command

**Figure 1:**
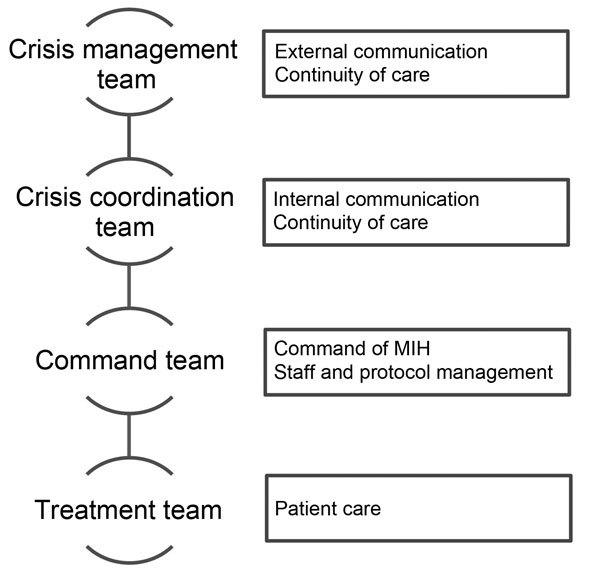
Planned command structure for potential admission of a patient with viral hemorrhagic fever, Major Incident Hospital (MIH), University Medical Centre of Utrecht, the Netherlands, 2014.

The chain of command was demarcated in 4 levels (Figure 1). First, the crisis management team would be responsible for external communication and coordination with the adjoining hospitals. Second, the crisis coordination team would operate between the crisis management team and the command team to ascertain continuity of care at UMC Utrecht. Third, the command team would oversee the admission of a patient to the MIH. Finally, the treatment team would provide medical treatment for the patient.

### Scenario Description and Routing

Flowcharts were developed that described 3 scenarios for routing a patient with suspected or confirmed VHF: 1) self-referral, 2) external referral, and 3) in-hospital referral from another ward. Security staff and a nurse would guide the patient to the MIH via a designated cleared route. Meanwhile, an emergency department nurse would open the MIH, activate the negative pressure system, and alert a team of trained nurses and an infectious disease specialist, all of whom would perform triage and assess the need to scale up the response.

**Figure 2:**
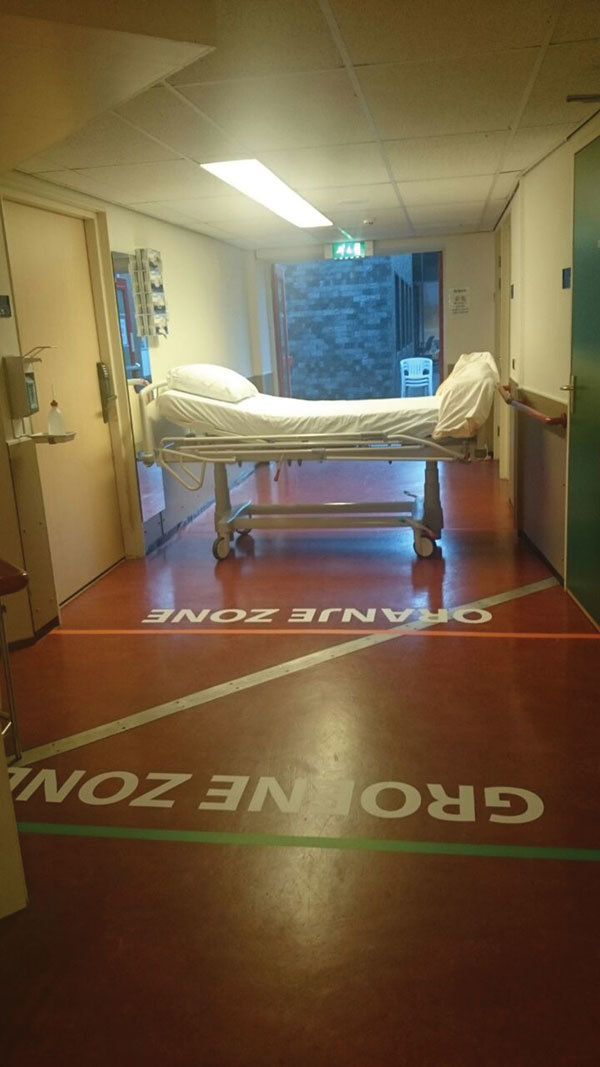
Entrance of isolation unit with demarcated zones, Major Incident Hospital, University Medical Centre of Utrecht, the Netherlands, 2014. Markings on the floor indicate a safe zone and potentially contaminated zones and delineate doffing zones (where potentially contaminated clothing and gear are removed) for ambulance and disinfection personnel.

VHF patients arriving by ambulance would enter the MIH through a separate entrance in the MIH. At this entrance, a 3-zone area was drawn on the floor to indicate the safe zone and potentially contaminated zones and to delineate doffing zones (where potentially contaminated clothing and gear are removed) for ambulance and disinfection personnel (Figure 2). Personnel from the appointed ambulance services also were trained in accordance with the revised protocols. 

### Infection Prevention Measures

#### Personal Protective Equipment

The selection of personal protective equipment (PPE) was based on national and international guidelines and tested for workability and comfort (*3*,*4*). A protective overall with a fluid-protected surgical gown combined with an FFP2 respirator (which filters >94% of airborne particles), a face shield, double-layer gloves, and double-layer foot protection would be worn over the standard surgery scrubs and clogs. Full-face masks conferring FFP3-level protection (i.e., filtering >99% of airborne particles) would be available for those performing high-risk procedures. PPE would be stocked to the extent that 1 patient could be treated for up to 14 days, and a list for backup suppliers and materials would be set up.

#### Medical and Other Equipment

In the patient’s room, disposable equipment would be used, such as cardboard pots with fluid-absorption granules for urine and feces, eating utensils, and other accessories. Exceptions would only be possible for items that could withstand final disinfection procedures after discharge.

#### Working Procedures

**Figure 3:**
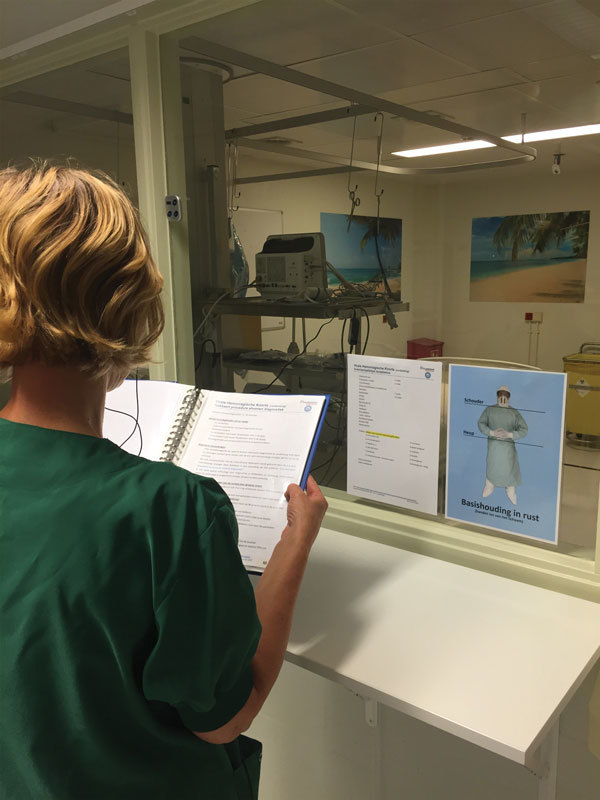
A buddy nurse demonstrates reading instructions in front of the isolation unit glass window for healthcare personnel working inside the unit, Major Incident Hospital, University Medical Centre of Utrecht, the Netherlands, 2014.

To ensure the safety of personnel, the “buddy system,” an extended version of the trainer-observer method (*4*), was introduced. In this system, a specialized nurse (buddy) guides and monitors all activities of the staff who are wearing PPE, starting with the donning of clothing and gear and ending with discarding all PPE. The buddy would be seated outside the unit in front of the glass window looking into an isolation room (Figure 3) and would guide the care provider in the room by reading aloud every step of the protocol being used, ensuring the minimization of risk behaviors arising from haste, stress, and the uncomfortably warm conditions felt while wearing PPE. The buddy and care provider would communicate by speakerphone inside the room connected to a mobile telephone. The glass windows would also facilitate monitoring of and communication with the patient. The maximum number of medical personnel present in an isolation unit was set to 1 at a time to ensure maximum safety. The maximum time spent in PPE was set at 45 minutes to minimize the loss of concentration caused by discomfort.

While a patient is in an isolation room, the nurses would work 8-hour shifts in teams of 3 persons (i.e., a bedside nurse, a buddy nurse, and a coordinating nurse). All procedures were summarized in task cards that would be used by the buddy to guide the bedside nurse. All protocols and task cards were made available through the hospital intranet.

#### Waste Management

Preparations for waste management were a major concern given the expected amount of waste and the time-consuming procedures involved (replacing a single waste container in the isolation unit can take as long as 20 minutes). Designated, sealable, 60-L waste containers would be used for waste storage, and waste management procedures were strictly protocolled and repeatedly conveyed through training.

In-hospital autoclave capacity appeared insufficient; therefore, waste destruction would be outsourced to an external facility. In accordance with transportation laws, one specific 20-L container had been approved for transport by public road (*5*). However, these containers were too small, and opening and closing them presented a safety risk. Therefore, category A medical waste (UN2814) containers were chosen; these were to be packed in a large plastic drum and the waste stored in a guarded and certified cooled sea container outside the hospital before transport.

#### Cleaning Procedures

The nursing staff were trained to perform the daily cleaning in the isolation unit. A limited number of cleaning staff were trained to perform the first disinfection after a patient transfer. For the final cleaning of the unit, an external company was contracted to perform disinfection with hydrogen peroxide treatment.

### Personnel

The required number of personnel was calculated for the admission of multiple patients to guarantee successful upscaling. During preparations, it proved necessary to activate the crisis coordination team to guarantee the availability of personnel from the hospital for frequent training sessions to ensure maximum availability during the admission of a patient. Flowcharts directed the alerting of in-house staff by team leaders during the acute phase; as necessary, a computerized alarm system would be activated to warn personnel by telephone.

**Figure 4:**
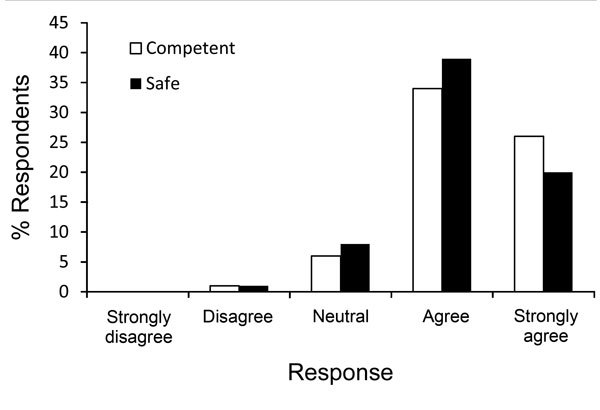
Results of a post-training survey conducted among staff indicating whether they felt competent and safe in caring for patients with Ebola, Major Incident Hospital, University Medical Centre of Utrecht, the Netherlands, 2014.

Some personnel were excluded from participation because of certain conditions (e.g., claustrophobia). Personnel were repeatedly trained in sessions of 1.5 hours, during which the donning and doffing of PPE, the buddy system, and other procedures were rehearsed (e.g., waste management, cleaning, and diagnostic procedures). These sessions were repeated every 10 weeks and resulted in a noticeable increase in the quality and safety of working conditions. The nursing staff of the MIH were prepared to fulfill the roles of buddy and trainer. A total of 126 staff members were trained (Table 1). After 2 training sessions, a survey conducted among personnel indicated that they felt sufficiently prepared (Figure 4).

**Table 1:** Personnel trained in preparation for admission of a patient with viral hemorrhagic fever, Major Incident Hospital, University Medical Centre of Utrecht, the Netherlands, 2014

Specialty/title	No. trained
Anesthetics specialist	1
Nurse trainer	13
Infectious disease specialist	8
Intensive care specialist	4
Internal medicine specialist	6
Nurse, emergency department	33
Nurse, intensive care unit	18
Nurse, infectious diseases	13
Pediatric infectious disease specialist	3
Pediatric intensive care specialist	5
Nurse, other department	11
Nurse, pediatric intensive care	5
Nurse, pediatrics	4
Resident infectious disease specialist	2
Total	126


Occupational health and safety service guidelines were developed for staff. All employees whose work involved contact with a VHF patient under protected conditions would be registered and required to monitor their temperature. In the event of unprotected contact with a VHF patient, personnel would be excluded from activities in the hospital and closely monitored by the public health service. If onset of VHF-associated symptoms occurred, personnel would be requested to contact the hospital’s occupational health and safety service and would be admitted to the isolation unit.

### Diagnostics

PCR testing for VHF was to be performed in 2 reference centers (the Erasmus Medical Center, Rotterdam, the Netherlands, and the Bernhard Nocht Institute for Tropical Medicine, Hamburg, Germany), which were appointed in accordance with national regulations and are in compliance with safety protocols (*6*,*7*). PCR testing was to be performed for EBOV, Marburg virus, Lassa virus, Crimean-Congo hemorrhagic fever virus, HIV, *Plasmodium* spp., and *Leptospira* spp. All materials were to be stored in plastic safety bags, placed in plastic containers, and then placed in cardboard shipping boxes (i.e., the “box-in-box” method).

Point-of-care laboratory tests were to be performed in the isolation unit by using I-STAT portable clinical analyzer (Abbott Point of Care, Inc., Princeton, NJ, USA). More extensive testing would be possible in one of the appointed external diagnostic centers.

A stethoscope equipped with a Bluetooth connection was acquired for auscultation of patients without physical contact. The radiology department was consulted to explore the possibilities of imaging in certain circumstances, such as the localization of a central venous catheter. However, these possibilities were limited by the confined space in the isolation rooms and by the need to decontaminate the equipment. It was then decided that the use of conventional radiography would not be possible. The option of a small portable ultrasound device was explored; however, the quality of the imaging was insufficient.

### Isolation Department and Adaptations

**Figure 5:**
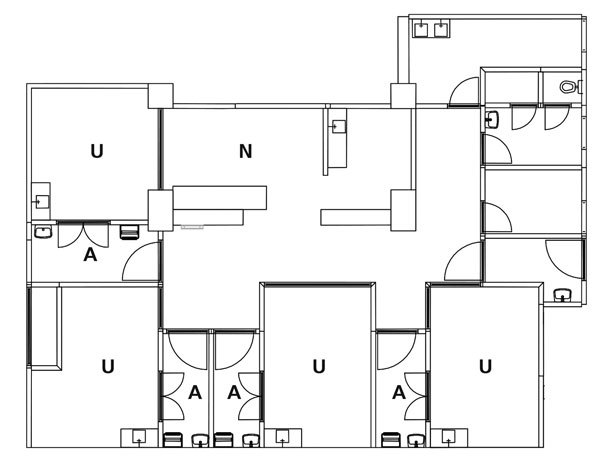
Schematic overview of the isolation department, Major Incident Hospital, University Medical Centre of Utrecht, the Netherlands, 2014. U, isolation unit; N, nursing station; A, access valve.

Only small adaptations to the isolation units were necessary. To prevent spread of the virus, running water taps were shut off, and sinks were disconnected from the sewage system. To ensure safety in cases of patient delirium and to enforce involuntary quarantine, the units were equipped with locks and safety glass. A schematic overview of the isolation department is shown in Figure 5.

### Medical Treatment Protocol

A medical protocol was developed to describe the standard diagnostic procedures and medical treatment according to the available guidelines. The protocol included preemptive treatment for malaria and administration of antimicrobial drugs for possible bacterial sepsis.

### Communication and Information

Flyers and banners were posted in the outpatient departments and in hospital entryways, requesting that patients, visitors, and personnel who had recently traveled to a high-risk area for EBOV infection inform hospital staff. Additionally, information was broadcast on a television screen in the emergency department entryway and waiting room. Staff at the emergency department and hospital wards were instructed to enquire actively about risk factors for VHF for patients with fever.

Local hospitals and other external partners were informed and advised about VHF procedures and referral to the MIH. Meetings to inform noninvolved hospital staff about EVD and the precautions taken by the hospital were organized and, before the arrival of an actual patient, repeated for the relatives of involved staff.

## Experiences during the Admission of an Ebola Patient

On December 4, 2014, the Ministry of Health, Welfare, and Sport requested that the MIH admit an EBOV-infected soldier who was residing in a hospital in Liberia. Arrival was planned for 2 days later. Daily updates about the health status of the patient and his transport allowed optimal preparation. The command structure functioned as outlined. Additional informational meetings were held for staff and relatives and were especially appreciated by the families of personnel, whose anxiety had been amplified by the increased media coverage of Ebola.

### Transfer and Admission

Before arrival of the patient, the transfer procedures were rehearsed twice with the involved ambulance and MIH personnel. Some changes had to be made to the infrastructure of the hospital entrance because of the differing PPE doffing procedures used by the designated ambulance services. It was then decided to involve no more than 2 ambulance services for future transport of VHF patients to the MIH.

At the time of patient admission, the command team, 4 trained nurses, and an infectious disease specialist were present, and intensive care unit (ICU) personnel and a cleaning team were on standby. Security personnel were present to prevent unauthorized persons from entering the unit and to prevent members of the news media from entering the hospital grounds.

The command team was continuously updated about the progress of the ambulance en route to the hospital. At 15 minutes before expected arrival, the first team of nurses started PPE donning procedures. The transfer of the patient at the hospital entrance was time-consuming because the patient was enveloped in a body bag, which resulted in more time required for triage by the first crew in PPE. The initial triage indicated no need for ICU admission. The initial diagnostics and medical treatment proved to be more time-consuming than expected. Cleaning and disinfection of the hospital entrance took >1 hour instead of the planned 45 minutes.

### Treatment 

All necessary medical and nonmedical activities (e.g., food delivery) were bundled to minimize the number of entries into the isolation room, resulting in 2–3 entries per 8-hour shift. Diagnostic blood samples were collected from an intravenous line to avoid high-risk procedures. Microbiological diagnostic procedures and sample transport to the diagnostic centers were supervised by the attending virologist and proceeded without incident. The results became available the same day. Coagulation tests were not available at the time; however, they were not required in this particular case.

Daily physician visits, except for replacement of the intravenous tube, were primarily conducted through the glass window by telephone. Spiritual counseling was provided at the request of the patient, and a tablet computer was provided for distraction and contact with family. The continuous presence of 3 nurses proved necessary during all 3 daily shifts. The availability of a coordinating nurse ensured that the interaction between the buddy and the bedside nurse was never disturbed. No safety incidents occurred. The limited working time of 45 minutes in PPE proved to be appropriate; however, an additional 20 minutes for recovery seemed to be warranted. PPE stock levels were always adequate. 

Waste production was lower than expected because of the relatively stable condition of the patient. The maximum number of 60-L barrels used was 8 on the first day and 3 on every following day. Guided transport of the barrels to the external facility was necessary only twice weekly. A complicating factor was the difficulty of appropriately closing the barrels in 5 instances, which necessitated the resealing of boxes inside other boxes before further transport.

### Discharge

After 6–7 days, all signs and symptoms of EVD in the patient had disappeared; however, the patient was dismissed from isolated treatment another 7 days later, after 2 PCR blood test results were negative for EBOV. The discharge procedure had been described only minimally in protocols and was developed in the days before dismissal. The isolation unit was sealed awaiting decontamination of the room by hydrogen peroxide gassing, which was performed 1 day after discharge. The isolation room was not made available until 14 days later because of the mandatory incubation period, the time required to interpret biostrips used to monitor the space, and the unavailability of staff from the external company during the end-of-year holidays. The entire isolation unit was unavailable for the admission of patients on the day of gassing because of interconnected air systems.

### Evaluation

All involved personnel were monitored daily for 3 weeks after their last shift, and none experienced onset of symptoms. The experiences of the admission were shared with other medical centers and the National Institute for Public Health. Revisions to the design of the isolation unit are under way and include the installation of automatic sliding doors and improvement of the communication equipment.

## Discussion

The EVD epidemic in West Africa during 2014–2015 underscored the need for hospitals worldwide to prepare for outbreaks of disease caused highly pathogenic and infectious organisms (*8*,*9*). During the fall of 2014, UMC Utrecht, which was already equipped with the MIH, intensified its preparations for the admission of VHF patients. These preparations proved to be time-consuming for all key players. In addition, the frequent training of staff led to scheduling complications; however, after activation of the crisis coordination team during the preparation phase, the sense of urgency increased, and departments were more motivated to provide staff.

Tracing procedures in the hospital resulted in increased alertness for VHF in patients with fever. Simulation exercises confirmed the value of protocols for triage and care and led to improvements of the procedures. Regular repetition was needed to sustain the level of alertness and knowledge of procedures. The value of protocols has been confirmed by the experience of other Western hospitals that have cared for patients with suspected or confirmed EBOV infection (*10*,*11*). However, data about preparedness and infection prevention measures are scarce; a single report about hospital preparations indicates that the trainer-observer method was used during PPE doffing and donning and that autoclaving took place at the hospital, but other procedures were not described (*12*).

The admission of an EBOV-infected patient was an opportunity to test all developed procedures. The initial transfer proved time-consuming and warrants further training with ambulance services. Moreover, because every regional ambulance service has slightly different PPE procedures, it proved appropriate to restrict the number of involved ambulance services for a single hospital.

The treatment of 1 patient was demanding on staff resources. Because the isolation unit was located outside the regular hospital wards, additional personnel were needed to staff the front office and to secure the cooling container on the premises. PPE use occurred without incident, but the discomfort caused by PPE was the largest complicating factor and warranted limiting the time spent in PPE to 45 minutes, with a 20-minute recovery period.

Minimal requirements for the selection of PPE had already been determined by national authorities on infection prevention. However, the selection of PPE differed among the UMC Utrecht and other hospitals in the Netherlands appointed for admission of Ebola patients; for example, some centers used rubber boots (instead of clogs), a hood with a powered air supply, or cooling vests. Although some of these options might provide more comfort, they are more costly and do not always provide additional safety. The buddy system was based on the trainer-observer method of donning and doffing PPE (*4*) but was extended to all high-risk procedures conducted in the isolation unit and ensured the safety and confidence of personnel. However, it was determined that the admission of >4 patients simultaneously should be avoided because the presence of multiple buddies in the isolation unit would compromise the audibility of instructions.

Waste destruction had to be outsourced because of the lack of autoclave capacity at the MIH, which led to additional costs and workload. Apart from the incidental failure to close a waste container, no safety incidents occurred.

Although the patient did not need care in the ICU, the admission increased awareness that the number of trained ICU staff would be insufficient to treat a patient in need of mechanical ventilation or hemodialysis for any extended period (*13*). The staffing required for these procedures would certainly lead to a restriction of available ICU beds for other patients. An intensified training program for ICU staff was devised, and more detailed ICU protocols are in development.

Although the absence of coagulation tests did not lead to a problem in this case, it might have in the case of a severely ill patient. Limited coagulation tests became available later. Also, the use of conventional radiography was deemed impossible; portable ultrasound devices are now being tested. A recent report underscores the need for advanced protocols to perform radiologic imaging in these circumstances (*14*).

During admission, protocols for discharge were still under development, leading to last-minute changes and some agitation among staff; however, discharge itself went well. The final cleaning procedure took longer than expected; on the day the patient was discharged, all 4 rooms were unavailable because the air treatment systems were interconnected. The system will therefore be adjusted (bifurcated) in the future. 

Communication was of utmost importance; not only did the hospital staff need information, but so did their relatives, who were concerned about the risks of working with Ebola patients. Also, the demand for extra security personnel was high because of the need to secure the stored waste and limit access to the MIH.

Although the isolation units in UMC Utrecht are located in a separate facility (the MIH), our experiences might be useful in other hospital settings (Table 2). Existing international and national protocols describe only the minimum requirements and therefore are not suitable for comparison. The preparations made and the lessons learned during the admission of an Ebola patient confirm the necessity of clear and practical protocols, a buddy system, and intensive staff training, all of which increase the safety of healthcare workers. Because of these measures, we experienced virtually no reluctance of personnel to be involved in the care of VHF patients. The demand on resources to treat VHF patients is high and can lead to understaffing at other departments at the expense of other patients. The availability of a dedicated major incident hospital has greatly increased the resources and preparedness of our center.

**Table 2:** Key lessons learned from admission of an Ebola patient, Major Incident Hospital, University Medical Centre of Utrecht, the Netherlands, 2014

Considerations for the future
• Protocols should be in place for all procedures.
• Limit the number of ambulance services eligible for patient transfer.
• The buddy system as extension from the trainer-observer role is invaluable in care for patients with viral hemorrhagic fever.
• Regular repetition of training is necessary.
• Time in personal protective equipment should be limited to 45 minutes, with an additional 20 minutes for recovery.
• The volume of biologic waste will be more than expected, and procedures for waste management need to be explored at an early stage.
• Remote, noncontact, sensors should be explored as possible tools in diagnostics.
• Specific engineering solutions are needed for every different infection scenario.
